# Functional and Sensory Properties of Pâtés Formulated with Emulsions from Chicken By-Products

**DOI:** 10.3390/foods14203488

**Published:** 2025-10-13

**Authors:** Zhanibek Yessimbekov, Eleonora Okuskhanova, Anuarbek Suychinov, Guldana Kapasheva, Baktybala Kabdylzhar, Assel Dautova, Alibek Muratbayev, Madina Jumazhanova

**Affiliations:** 1 Kazakh Research Institute of Processing and Food Industry (Semey Branch), Semey 071410, Kazakhstan; zyessimbekov@gmail.com (Z.Y.); asuychinov@gmail.com (A.S.); gena.89.89@mail.ru (G.K.); baktybala.20@mail.ru (B.K.); 2 Research School of Food Engineering, Shakarim University, Semey 071412, Kazakhstan; aska_nur@mail.ru (A.D.); mr.muratbayev.a@gmail.com (A.M.); madina.omarova.89@mail.ru (M.J.)

**Keywords:** chicken by-products, protein–fat emulsion, fat replacer, pâté formulation, functional properties, lipid oxidation, organoleptic quality

## Abstract

This study evaluated the potential of chicken by-products (hearts, gizzards, and skin) as functional raw materials for protein–fat emulsions to partially replace animal fat in pâtés. Five variants of pâté (PV1–PV5) were prepared, including a control without emulsion and four samples with increasing emulsion levels. Emulsions were formulated from chicken by-product mixtures and vegetable oil with potato starch, sodium bicarbonate, and salt to improve solubility and viscosity. The chemical composition of by-product mixtures varied with organ ratio: heart-rich mixtures supplied higher protein, supporting emulsion stability, whereas skin-rich mixtures contributed more fat for texture. Emulsion composition ranged from 6.6–8.1% protein, 19.1–28.4% fat, and 56.7–66.9% moisture. Functional properties depended on formulation balance: water-holding (58–67%), fat retention (70–83%), emulsifying capacity (50–62%), and stability (47–55%). Variant 5 achieved the most favorable combination of composition, stability, and viscosity. In pâtés, emulsion addition reduced protein and fat but increased ash and carbohydrate contents (*p* < 0.05), improving hydration and stability. Fat retention rose up to 83% and emulsion stability up to 62%. Drip loss declined markedly from 9.2% in the control to 3.6% in Variant 5, while yield stress decreased by 25%, producing softer, more spreadable products. Sensory evaluation favored emulsion-containing samples, with PV-5 scoring highest in texture and appearance. TBARS values rose with the amount of emulsion due to higher PUFA, but acid numbers increased more slowly, indicating reduced hydrolytic rancidity. Overall, pâté with 25% of emulsion offered the best balance of technological performance, sensory quality, and lipid stability, highlighting chicken by-products as sustainable emulsifiers in pâté production.

## 1. Introduction

The poultry sector is one of the fastest-growing segments of the meat industry and relies on integrated processing chains to convert whole carcasses into a wide range of products with consistent quality and safety. During poultry processing, besides the main meat cuts, many secondary by-products are produced. These by-products have good nutritional value but are often not fully used [1,2]. Better use of them is important today in meat science because it can improve product quality, make processing more efficient, reduce costs, and support sustainable production.

In poultry processing, edible by-products make up an important part of the total yield. These by-products include different tissues that have their own biochemical composition and functional properties. Of particular relevance to the present work are chicken hearts and gizzards. Chicken hearts are predominantly muscular tissue, supplying easily digestible protein together with minerals (e.g., iron, zinc, copper) and B-group vitamins. Their typical proximate profile (about 15% protein and 10% fat, wet basis) suggests potential both as a protein contributor and as a moderate lipid source [3,4]. Gizzards are likewise nutrient-dense, containing approximately 20% protein and 4–6% fat, and provide thiamine, niacin, and folate. Their relatively low energy content and balanced amino-acid composition make them attractive for formulating higher-protein, lower-calorie products [5]. Chicken skin, in contrast, is abundant and rich in lipids and collagenous proteins. While its high fat and connective-tissue content can limit direct use in conventional formulations, it offers a readily available source of functional lipids and structural proteins when appropriately processed [6,7].

Rational utilization of these by-products addresses both economic and societal priorities. From an economic perspective, redirecting nutrient-rich tissues from feed or low-value outlets into human food increases carcass value and reduces reliance on high-cost prime meat. From a processing point of view, the diversity of by-products can be used as an advantage. Each tissue has different useful properties: proteins from hearts improve emulsification and gel formation [8]; skin provides collagen and lipids that add smoothness and enhance texture [9]; and the high protein content of gizzards increases water binding [10]. This type of valorization also supports the aims of reducing waste and efficiently using resources, which expands the range of protein-dense foods.

A proven route to integrate by-products into meat matrices is through structured emulsions. Protein–fat (oil-in-water) emulsions are multicomponent dispersed systems in which the fat phase is stabilized by surface-active proteins and polysaccharides within an aqueous continuous phase [11]. When designed for use in comminuted meat products, such systems can increase water and fat retention, lower cooking losses, and improve texture and consistency. Myofibrillar proteins are especially effective in this role, as they unfold and adsorb at the oil–water interface to form viscoelastic interfacial films, thereby stabilizing lipid droplets [12]. Polysaccharides and starches further contribute by thickening the continuous phase, immobilizing water, and limiting droplet mobility [13].

Emulsions are important because they allow partial replacement of animal fat with vegetable oil, improving the fatty-acid profile while preserving sensory qualities such as texture and sliceability [14]. Processing conditions are equally important for emulsion quality. High-shear comminution decreases droplet size, creating greater surface area for protein adsorption and enhancing interfacial stability. During thermal processing, starch gelatinization reinforces the protein network and traps aqueous and lipid phases, minimizing separation [15]. These mechanisms result in a coherent gel that underpins desirable technological properties.

Emulsion functionality is commonly evaluated through water-holding capacity (WHC), fat retention, cooking yield, textural performance, and oxidative stability. WHC is critical because it determines how much moisture remains bound during storage and heating, directly influencing juiciness, yield, and product stability [16]. Fat retention prevents fat-out and ensures consistent texture, while cooking yield reflects the overall capacity of the emulsion to withstand heat without losses. Smaller, protein-coated droplets also influence oxidative stability, though incorporation of polyunsaturated oils may increase susceptibility to lipid peroxidation if not balanced with antioxidants [17]. Emulsions serve not only as structural systems but also as determinants of functional, nutritional, and sensory quality in meat products. These principles form the scientific basis for developing chicken by-product-based emulsions designed to enhance pâté formulations.

This study aims to evaluate the potential of emulsions derived from chicken by-products as functional fat replacers in pâtés, focusing on their effects on technological performance, nutritional composition, and sensory acceptability.

## 2. Materials and Methods

### 2.1. Samples

Chicken meat and edible by-products were supplied by the poultry processing plant “Vostok-Broiler” (Semey, Kazakhstan). All raw materials were transported to the laboratory under controlled chilled conditions using portable insulated containers at a temperature of approximately +4 °C to ensure microbiological safety and preservation of functional properties. Upon arrival (after 15 min), materials were immediately inspected for freshness and integrity before further processing. Refined vegetable oils and other supplementary ingredients (potato starch, table salt, sodium bicarbonate, and spices) were purchased from the local supermarket “ADAL” (Semey, Kazakhstan). All ingredients were food-grade and used without further modification.

### 2.2. Preparation of Chicken By-Product Mixture

Before experimental preparation, the proportions of chicken skin, gizzards, and hearts were mathematically modelled to predict balanced protein and fat ratios, ensuring optimal texture and emulsifying potential. These calculated formulations were then prepared and verified experimentally as shown in Table 1.

Raw materials (chicken skin, gizzards, and hearts) were received under refrigeration (≤4 °C), visually inspected for foreign matter, defects, and contamination, and processed the same day. Gizzards were trimmed to remove the internal yellow cuticle and residual contents and rinsed thoroughly with potable running water at 10–15 °C. Hearts were freed of visible blood, clots, and large vessels, with excess surface fat trimmed, and then rinsed; skins were checked for residual feathers and debris and rinsed similarly. Prepared tissues were drained in perforated containers for 5–10 min to remove free water. Each tissue was then ground through a sanitized meat grinder fitted with a 3–5 mm plate, with product temperature controlled to ≤10 °C to minimize protein denaturation. Equipment was chilled and cleaned between tissues. Four by-product mass formulations were prepared by weight using a calibrated laboratory balance (±0.1 g) and placed in numbered containers.

For each variant, the preground components were transferred to a stationary bowl-cutter or high-shear blender and mixed with concurrent fine size reduction for 1–2 min to obtain a homogeneous paste; the mass temperature was monitored continuously and maintained ≤12 °C. The appearance, consistency, and color of each paste were recorded immediately after mixing. Each batch was labeled with variant code, date, and time, packed in airtight food-grade containers, and held under refrigeration at 2–4 °C for no longer than 12 h prior to subsequent analyses and use in emulsion preparation aimed at screening chemical composition and functional–technological properties (water- and fat-retention, emulsifying capacity, emulsion stability).

### 2.3. Preparation of Emulsion

The by-product mass included poultry skin as a natural source of structural fat. However, for emulsion preparation, an additional free lipid phase was required to ensure stable oil-in-water droplet formation. Refined vegetable oil was incorporated as a readily dispersible fat source. Its inclusion ensured control of the fat-to-protein ratio, promoted droplet stability under shear, and improved the unsaturated fatty acid profile of the emulsions. Eight oil-in-water emulsions were prepared with different ratios of by-product mass, vegetable oil, water, and starch. Before processing, the by-product mass was chilled to 2–4 °C. All ingredients (refined vegetable oil, potable water, potato starch, sodium bicarbonate, and sodium chloride) were weighed on a calibrated balance and allocated to individually labeled containers (Figure S1). The formulations are shown in Table 2.

For each batch, ingredients were loaded into a chilled bowl-cutter or high-shear blender in the following sequence to promote protein extraction and interfacial film formation: by-product mass, salt (uniformly dispersed over the surface), sodium bicarbonate, dry starch, approximately half of the total water to wet the matrix, vegetable oil added in a fine stream to facilitate droplet breakup, and finally the remaining water to reach target solids. Mixing began at medium speed for 20–30 s to pre-disperse components, followed by high-speed comminution for 60–90 s with intermittent scraping of the vessel wall; product temperature was monitored continuously and maintained ≤12 °C to avoid protein denaturation. Emulsion readiness was defined as a smooth, homogeneous paste with no visible starch clumps or oil streaks; if heterogeneity was observed, short additional bursts (15–20 s) of high-speed mixing were applied while holding temperature limits. Each finished emulsion was transferred to a clean food-grade container with a tight-fitting lid, labeled with variant code, date, and time, and held under refrigeration at 2–4 °C for no longer than 12 h before subsequent physicochemical and functional testing. Visual homogeneity was recorded for all batches as an in-process quality check.

### 2.4. Preparation of Pâté

All ingredients are received under refrigeration (≤4 °C), inspected for integrity, odor, and temperature, and processed the same day (Table 3, Figure S2). Chicken livers are trimmed to remove connective tissue and bile ducts, rinsed in cold potable water, soaked at 2–4 °C (1:2, *w*/*w*; 20–30 min), blanched at 90–95 °C for 2–3 min, rapidly cooled under running cold water, drained, and comminuted through a 3 mm plate. Deboned poultry meat is trimmed of cartilage and surface fat and ground through a 3 mm plate. Onions are peeled, briefly soaked in cold water (5–10 min), sautéed to translucency (≈110–120 °C, 3–5 min), cooled, and minced; carrots and parsley root are peeled, cut, and either blanched (90–95 °C, 3–5 min) or lightly sautéed (2–3 min), then cooled and minced. The protein–fat emulsion is prepared according to the selected formulation by blending the previously milled by-product mass with vegetable oil, starch, sodium bicarbonate, water, and salt in a jacketed cutter under chilled conditions (≤10 °C) to obtain a homogeneous oil-in-water system; the emulsion is held at 0–4 °C until use (Figure S3).

The pâté batter is assembled in a paddle mixer or high-capacity blender by combining ground poultry meat, prepared liver, the designated emulsion proportion, beef fat where applicable, processed onions, carrots, parsley root, spices, salt, and broth; mixing proceeds at ≤10 °C with intermittent scraping until a uniform paste is obtained. The batter is filled into sanitized No. 4 containers (250 g net) with standardized headspace (≈5–10 mm) and closed with sterilized lids using a semi-automatic seamer. Sealed containers are thermally processed in a steam autoclave at 112 °C and 0.08 MPa using a 20–65–20 min schedule (come-up, holding, and pressure-assisted cooling), with core temperature verified in pilot units (Figure S4). Packs are cooled to ≤30 °C in-vessel, then to ≤20 °C by air or water, rinsed, dried, inspected (seam integrity, vacuum, visual defects), coded/labelled, and transferred to refrigerated storage (2–4 °C) pending analysis.

### 2.5. Determination of Protein Content

The protein content was determined according to GOST 25011-2017 [18] using the Kjeldahl method. This method involves mineralization of organic matter followed by nitrogen quantification from the amount of ammonia formed. The protein mass fraction was calculated using the standard formula provided in [17].

### 2.6. Determination of Fat Content

The fat content was determined according to GOST 23042-2015 [19] using Soxhlet extraction. Dried samples were extracted with petroleum ether, and the solvent was removed before drying the recovered fat to constant weight.

### 2.7. Determination of Water Content

The method is based on drying the analyzed sample with sand to a constant mass at a temperature of (103 ± 2) °C for 120 min.

### 2.8. Determination of Ash Content

Ash content was determined according to GOST 31727-2012 [20]. Samples were dried, charred, and ashed at 550 °C in a muffle furnace until constant weight was achieved. The ash fraction was calculated gravimetrically from the residue.

### 2.9. Determination of Carbohydrate Content

The carbohydrate content was determined by difference using Formula (1):

(1) Carbohydrates, % = 100 − (Moisture + Portein + Fat + Ash)

### 2.10. Determination of pH

The active acidity (pH) was measured according to ST RK ISO 2917-2009 [21] using a calibrated pH meter Seven2Go (Mettler Toledo, Greifensee, Switzerland). A homogenized water extract (1:10, sample–distilled water) was prepared, and pH was recorded at 20 °C after calibration with standard buffers.

### 2.11. Determination of Water Activity

Water activity (a_w_) was determined using an aWLife instrument (Steroglass, Perugia, City, Italy) according to the manufacturer’s protocol. Prior to analysis, the device was calibrated with standard solutions to ensure accuracy. Prepared samples were placed into the measurement chamber, equilibrated at 25 °C, and the a_w_ value was automatically recorded once equilibrium was reached. Calibration and optimization functions were applied regularly to maintain measurement reliability.

### 2.12. Determination of Water-Holding and Fat-Retention Capacities

Water-holding and fat-retention capacities were determined according to [22]

### 2.13. Determination of Emulsifying Capacity and Emulsion Stability

The emulsifying capacity of samples was determined according to a standardized protocol. A sample (7 g) was dispersed in 100 mL of distilled water using a laboratory homogenizer at a shear rate of 66.6 s^−1^ for 60 s. Subsequently, 100 mL of refined sunflower oil was added, and the mixture was homogenized at 1500 s^−1^ for 5 min to obtain a uniform emulsion. The emulsion was transferred into four calibrated centrifuge tubes (50 mL each) and centrifuged at 500 s^−1^ for 10 min. After centrifugation, the volume of emulsified oil was measured. The emulsifying capacity (EC, %) was calculated using the following Formula (2):

(2) $$EC=\frac{V_{1}\cdot 100}{V}$$

where

*V *_1_—volume of emulsified oil (mL),
*V*—total volume of added oil (mL).

Emulsion stability was assessed by subjecting the emulsion to thermal treatment. Samples were heated at 80 °C for 30 min, followed by rapid cooling in water for 15 min. The emulsions were then transferred into four calibrated centrifuge tubes (50 mL each) and centrifuged at 500 s^−1^ for 5 min. The volume of the emulsified layer was determined.

Emulsion stability (ES, %) was calculated according to Formula (3):

(3) $$ES=\frac{V_{1}\cdot 100}{V_{2}}$$

where

*V *_1_—volume of emulsified oil after centrifugation (mL),
*V *_2_—total volume of emulsion (mL).

This method quantifies both the emulsifying ability of the protein fraction to bind oil at the interface and the resistance of the emulsion to phase separation under thermal and centrifugal stress [23].

### 2.14. Microstructural Analysis

The microstructure of pâté samples was analyzed according to the standard procedure outlined in GOST 19496-2013 [24]. For each variant, at least three units of product were sampled. Samples were fixed in 10% neutral buffered formalin at 22 ± 1 °C for 24 h and stored in the same solution until analysis. Before sectioning, fixed samples were rinsed with cold running water for 15 min. Loose samples were pre-embedded in gelatin (12.5% for 6 h, then 25% for 12 h at 37 °C, followed by cooling at 5 ± 1 °C) to improve section stability. Sections of 10–30 μm thickness were prepared on a freezing microtome and mounted onto pre-treated glass slides. General staining was carried out with Ehrlich’s hematoxylin (3–4 min), differentiated in 1% HCl water, blued in 1% ammonia solution, and counterstained with 1% eosin. Prepared slides were mounted in glycerin–gelatin medium without dehydration. Microscopic evaluation was performed in transmitted light using an MBS-100T microscope (BioLab, Ningbo, China) with objectives up to 40×, with identification of muscle, connective, fat, epithelial, and plant components. Structural integrity, droplet distribution, and evidence of protein or fat destabilization were recorded, and a semi-quantitative assessment of component prevalence was applied when necessary.

### 2.15. Determination of Emulsion Viscosity

The dynamic viscosity of the samples was measured using a BOYN Digital Viscometer (BOYN Instrument Co., Ltd., Shanghai, China) according to the manufacturer’s instructions. A 250 mL thermostated beaker was filled with 100 mL of the sample and equilibrated to a temperature of 18–22 °C, monitored with a precision thermometer (±0.2 °C). The measuring spindle was immersed until the reference mark was covered by the sample, after which a constant rotation speed of 6 rpm was applied. Readings displayed by the instrument were recorded after 60 s of operation.

### 2.16. Determination of Yield Stress of Pâté Variants

Yield stress was determined using the universal texture analyzer Structurometer ST-2 (Radius, Moscow, Russia) by the method described in [25].

### 2.17. Determination of Drip Loss

Drip loss of pâté variants was measured as the proportion of liquid released after thermal processing. Approximately 50 g of each pâté sample was weighed into glass jars, sealed, and subjected to sterilization under standard processing conditions. After cooling to room temperature, the exuded liquid was carefully decanted and weighed. Drip loss (%) was calculated as the ratio of released liquid mass to the initial sample mass, multiplied by 100 [26]. All measurements were carried out in triplicate.

### 2.18. Organoleptic Evaluation of Canned Pate

The sensory properties of canned pâté were assessed in accordance with the guidelines of GOST 33741-2015 [27]. A panel of seven trained assessors from the Semey branch of the Kazakh Research Institute of Processing and Food Industry carried out the evaluation. Before tasting, the cans were inspected for external quality, including integrity of the container, absence of leaks, deformation, or corrosion. Samples were heated in a water bath for 20 min before opening. Sensory assessment followed a standardized sequence: appearance, color, odor, consistency, and taste. Appearance was judged by the uniformity of chopping, shape retention, the state of the broth, and the absence of foreign inclusions. The trained tasting panel visually examined the color under standardized daylight conditions, comparing each sample against the typical appearance expected for this product type (uniform color, absence of dark or grayish tones, and consistency between surface and internal color). Odor was assessed for typicality and harmony, and the presence of off-odors was recorded. Consistency was characterized in terms of tenderness, fibrousness, uniformity, chewability, and presence of coarse or rigid particles. Taste was examined for typical flavor, overall acceptability, and the absence of undesirable or extraneous flavors.

### 2.19. Determination of Total Viable Count (TVC)

The total viable count (TVC) was determined following standard microbiological procedures. For each sample, 10 g was aseptically homogenized with 90 mL of sterile physiological saline (0.85% NaCl) to prepare a 10^−1^ dilution. Serial tenfold dilutions were then prepared as required. Aliquots of 0.2 mL from appropriate dilutions were inoculated onto Petritest™ substrates (“Alternativa” Company, Saratov, Russia) and incubated at 36 ± 1 °C for 18 h. After incubation, colonies were enumerated, and TVC was expressed as colony-forming units per gram (CFU/g), taking into account the respective dilution factors. All determinations were performed in triplicate, and mean values were used for analysis [28].

### 2.20. Determination of the Thiobarbituric Acid Reactive Substances

Thiobarbituric acid reactive substances (TBARS) values and acid numbers were determined in pâté variants during short-term storage. Immediately after thermal processing and cooling, at least three cans from each formulation were opened under aseptic conditions. The contents were homogenized, and samples were taken for initial analysis (Day 0). The remaining pâté in the opened cans was covered, maintained under refrigeration at +2 to +4 °C, and sampled daily for five consecutive days. At each interval, subsamples were withdrawn and analyzed for thiobarbituric acid-reactive substances (TBARS, expressed as mg MDA/kg) and acid value (expressed as mg KOH/g fat), providing a time-course assessment of lipid oxidation and hydrolysis during refrigerated storage.

Lipid oxidation was assessed by measuring TBARS according to GOST R 55810 [29]. The method is based on the reaction of malondialdehyde (MDA), formed during oxidation of unsaturated fatty acids, with thiobarbituric acid (TBA) to produce a pink chromogen measured spectrophotometrically at 535 ± 10 nm. The analytical range is 0.039–2.000 mg MDA/kg of product.

For analysis, homogenized samples were treated with hydrochloric acid, and MDA was released by distillation. The distillate was reacted with TBA solution in a boiling water bath for 35 min, then cooled, and the absorbance was recorded. TBARS concentration (X, mg MDA/kg) was calculated using Formula (4):

*X* = *A* × 7.8 (4)

where *A* is the measured absorbance,

7.8 is a constant conversion factor relating absorbance to MDA concentration.

### 2.21. Studying Acid Number Development in Pâté

The acid number (AN) of pâté samples was determined titrimetrically in accordance with GOST R 55480 [30]. A homogenized portion of 5–10 g was transferred into a conical flask and extracted with 50 mL of neutralized ethanol–diethyl ether solution (1:1, *v*/*v*) by mixing for 5 min. The mixture was then heated in a water bath at 50–60 °C for 10 min to facilitate the release of free fatty acids. After cooling to room temperature, 2–3 drops of phenolphthalein indicator were added. The solution was titrated with 0.1 M sodium hydroxide (NaOH) until a stable pink coloration persisted for at least 10–15 s. The volume of NaOH consumed was recorded and used to calculate AN, expressed in mg KOH per g of sample, according to Formula (5):

(5) X=V·K·5.61m

V—volume of 0.1 mol/dm^3^ potassium hydroxide solution consumed for titration, cm^3^;
K—correction factor to the nominal concentration of solutions;
m—mass of fat in the analyzed sample, g;
5.61—mass of potassium hydroxide corresponding to 1 cm^3^ 0.1 mol/dm^3^ of potassium hydroxide solution, mg.

### 2.22. Statistics

Each pâté variant was produced in a batch of 10 cans (250 g each), totaling 50 cans. From these, three cans per variant were designated for chemical and functional analyses and used as biological replicates, with each parameter measured in technical triplicate. All experiments were performed in triplicate, and mean values with standard deviations were calculated for each parameter. Statistical analysis was conducted using Excel 2016 (Microsoft Corporation, Redmond, WA, USA) and Statistica 12 PL (StatSoft, Inc., Tulsa, OK, USA). A one-way analysis of variance (ANOVA) was applied to evaluate differences among experimental variants and the control. A two-way ANOVA was performed to assess the interaction between formulation and storage period. When the ANOVA indicated significance, Tukey’s Honest Significant Difference (HSD) test was used for pairwise comparisons between group means. Differences were considered statistically significant at *p* ≤ 0.05.

## 3. Results

### 3.1. Chemical Composition of Chicken By-Products

The chemical composition of the selected chicken by-products was analyzed to determine their potential as raw materials for the preparation of by-product masses (Table 4). The results showed that the heart contained 75.88% moisture, 16.30% protein, 6.22% fat, and 1.6% ash. The gizzard exhibited a similar moisture content (75.54%) but was characterized by the highest protein fraction (18.59%) and comparatively low fat (4.57%) and ash (1.3%). In contrast, chicken skin exhibited a distinct profile, characterized by significantly lower moisture (59.72%) and elevated fat content (24.46%), while its protein content was moderate (15.06%), and its ash content was the lowest (0.76%).

These differences in proximate composition are significant for the formulation of protein–fat emulsions. The heart, with balanced protein and moderate fat levels, can provide both structural support and a source of lipids. The gizzard, as the most protein-rich component, is expected to contribute strongly to emulsifying capacity and water-binding, which are critical to the stabilization of multiphase systems. The skin, by contrast, represents a concentrated lipid source capable of increasing energy value and enhancing spreadability, but its high fat fraction requires sufficient protein co-components to ensure stable incorporation into emulsions [31].

Independent characterizations of chicken by-products confirm these roles. Seong et al. (2015) reported that edible offal typically contains 11–18% protein and 0.8–4.5% fat, with gizzard and liver at the upper end of the protein range, while hearts supply both protein and essential micronutrients [32]. That study also highlighted favorable lipid quality, with polyunsaturated-to-saturated fatty acid ratios ≥ 0.4, alongside elevated levels of minerals such as iron, zinc, and manganese, and B-group vitamins. These findings support the nutritional rationale for selecting undervalued viscera such as heart and gizzard as functional protein sources in emulsion systems, in line with the protein-rich and moderate-fat profiles determined in the present work.

Overall, the obtained data emphasize that none of the individual by-products alone provides the optimal composition for pâté formulation. Instead, strategic blending of heart, gizzard, and skin is necessary to combine their complementary properties. The chemical profiles demonstrate that the gizzard is most suitable for protein enrichment, the heart for balance, and the skin for fat contribution, laying the foundation for selecting appropriate ratios in the next experimental stage.

### 3.2. Chemical Composition of By-Product Mixture

The proximate composition of the four experimental by-product mixture variants reflects the influence of different proportions of heart, gizzard, and skin on nutrient balance and potential technological functionality (Table 5). Variant 1 demonstrated the highest protein concentration (17.9%) with moderate fat (11.88%) and ash (2.51%), while maintaining a relatively low moisture content (67.71%). This suggests a favorable ratio for protein availability, which is critical for emulsification, and sufficient lipid levels to support texture development in a protein–fat emulsion. Variant 2, in contrast, contained the highest fat fraction (14.93%) and slightly lower protein (15.51%), with moisture comparable to Variant 1 (67.11%). The elevated lipid concentration in this formulation increases caloric density and contributes to softness, but the reduction in protein may weaken structural stability unless additional emulsifiers are introduced.

Variant 3 presented a balanced profile with protein at 16.07% and the lowest fat among all options (9.96%), accompanied by high moisture (71.94%). This composition suggests that it could favor water retention and juiciness, although the lower lipid fraction may limit spreadability and richness in pâté formulations. Variant 4 showed the lowest protein level (14.71%) with moderate fat (10.37%) and the highest moisture (72.82%). Such a profile indicates a dilution of protein functionality, potentially reducing emulsifying capacity, though the higher water fraction could enhance tenderness.

Taken together, the data emphasize that composition strongly shifts with by-product ratio, impacting protein-to-fat balance. Variants 1 and 2 appear most promising: Variant 1 for its higher protein supporting emulsion stability, and Variant 2 for its fat enrichment contributing to product mouthfeel. These findings underline the importance of aligning raw composition with the targeted technological role in the pâté emulsion system.

### 3.3. pH and Water Activity of By-Product Mixture

**Figure 1:**
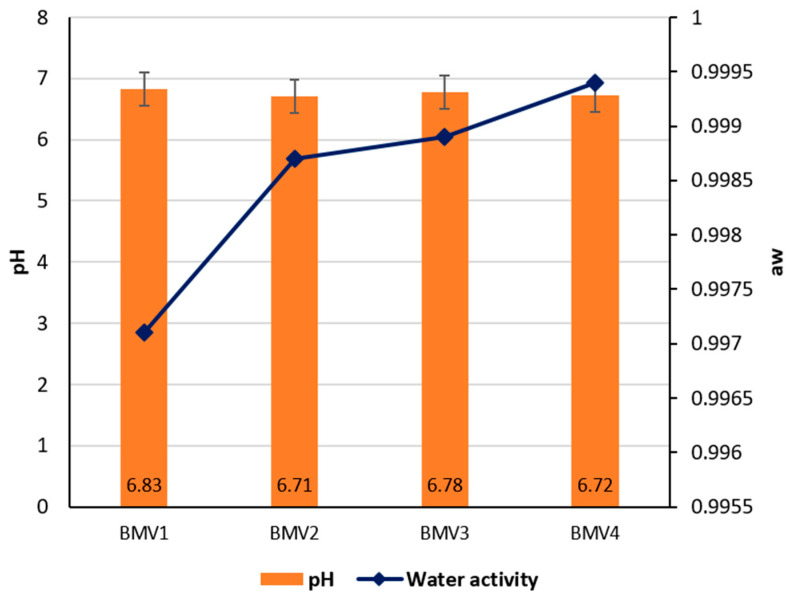
pH and water activity of by-product mixture.

The pH and water activity values were similar among the by-product mixture variants, yet small variations were present that could affect their technological behavior. Variant 1 exhibited the highest pH value (6.83) and the lowest water activity (0.9971), a combination suggesting slightly more favorable protein functionality and marginally reduced microbial risk compared to other formulations (Figure 1). Variant 2 displayed the lowest pH (6.71) alongside a relatively high water activity (0.9987), conditions that may enhance microbial susceptibility while reducing protein solubility, which could negatively influence emulsion stability. Variant 3 had a moderately high pH (6.78) with water activity at 0.9989, representing an intermediate state that balances protein reactivity and hydration but without pronounced stability advantages. Variant 4 demonstrated the lowest stability indicators, with a pH of 6.72 and the highest water activity (0.9994), reflecting a more diluted system prone to microbial growth and weaker protein–protein interactions.

Overall, the results suggest that Variant 1 provides the most suitable chemical environment for emulsion formation, as its higher pH may promote protein solubility and emulsifying capacity, while the lower water activity could contribute to improved safety and storage stability. From a scientific perspective, pH strongly influences the charge distribution on myofibrillar proteins, thereby regulating their water-binding and emulsifying potential [33]. Meanwhile, water activity reflects the proportion of free water available for microbial growth and chemical reactions; even small differences near unity can significantly affect product stability and shelf life [34].

### 3.4. Functional–Technological Properties of By-Product Mixture

**Figure 2:**
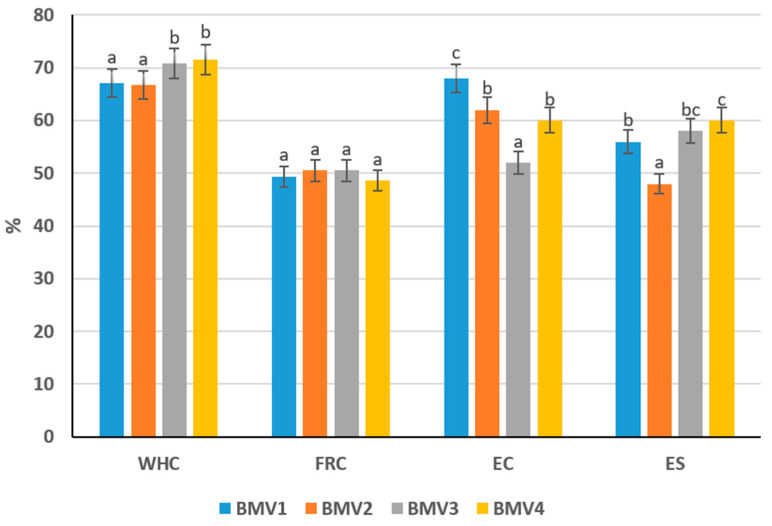
Functional–technological properties of by-product mixture. Different lowercase letters (a–c) indicate statistically significant differences within the same indicator (*p* < 0.05).

The evaluation of water-holding (WHC), fat retention (FRC), emulsifying capacity (EC), and emulsion stability (ES) demonstrated clear differences among the four by-product mass variants, which are critical for predicting their performance in pâté emulsions (Figure 2). The capacity of meat to retain its own water and any added moisture, often described as water-holding capacity (WHC), is a central quality factor in comminuted and cooked systems. It directly determines how much water remains bound during storage and heating, thereby influencing juiciness, yield, and textural attributes [35]. Inadequate WHC leads to higher cooking losses, weaker texture, and reduced sensory acceptance, as well as lower process efficiency.

Variants 3 and 4 displayed significantly higher WRC values (70.78% and 71.49%) compared with Variants 1 (67.05%) and 2 (66.73%) (*p* < 0.05). This indicates that gizzard- and heart-rich mixtures provide stronger hydration and water-holding ability, which is advantageous for minimizing cooking losses and maintaining juiciness in emulsified products. Salt plays an additional role in this process, since ionic interactions between salt ions and proteins enhance hydration. This so-called “salting-in” effect not only improves protein solubility but also increases water binding, meaning that moderate salt addition can simultaneously improve flavor, extend shelf life, and reinforce emulsion stability [36].

The highest fat retention was observed in Variants 2 and 3 (both 50.5%), exceeding the values of Variants 1 (49.4%) and 4 (48.6%), though the differences remained relatively small and not statistically significant (*p* > 0.05). This suggests that the presence of gizzard or skin contributes to lipid stabilization, an important factor in reducing fat separation during thermal processing.

Variant 1 showed the greatest EC (68%), significantly higher than Variants 3 (52%) and 4 (60%) (*p* < 0.05). This result is consistent with its elevated protein fraction, as proteins are key surface-active agents in emulsions. Variants 2 and 4 exhibited intermediate EC values (62% and 60%), while Variant 3 was markedly less effective, suggesting protein quality rather than absolute content influences emulsification efficiency.

Emulsion stability reflects the ability of the dispersed system to resist breakdown and maintain its structure over time. It is one of the key goals in formulating meat emulsions, since a stable system ensures minimal phase separation and consistent product quality. Stability was highest in Variant 4 (60%) and Variant 3 (58%), both significantly greater than Variant 2 (48%) (*p* < 0.05). Variant 1 showed intermediate stability (56%), indicating that despite strong emulsification capacity, stabilization against phase separation may require additional structural support.

The data confirm that compositional differences among by-product masses translate into measurable functional disparities. Protein-rich formulations (Variant 1) favor emulsifying ability, whereas heart-dominant or gizzard-rich blends (Variants 3 and 4) enhance water binding and emulsion stability, attributes essential for pâté quality. In contrast, Variant 2, although rich in fat and FRC, underperformed in stability, suggesting limited suitability without formulation adjustments. These findings highlight the necessity of selecting by-product ratios that balance protein functionality with water and fat retention to optimize pâté emulsions.

Based on the comparative analysis of chemical composition and functional–technological properties, the most preferable by-product mass for use in protein–fat emulsions is Variant 4, which contains 25% chicken skin, 25% gizzard, and 50% heart. This formulation demonstrated the highest water retention capacity (71.49%) and emulsion stability (60%), both of which are essential for maintaining product consistency, reducing syneresis, and ensuring shelf-life stability in pâté systems. Although its protein content (14.71%) was lower than in other variants, the heart-rich composition provided a balanced matrix that allowed effective hydration and structural integrity. Its moderate fat content (10.37%) also leaves formulation flexibility for the incorporation of vegetable oil without exceeding lipid saturation. Compared with other formulations, Variant 4 showed the most stable performance under processing conditions, indicating that it provides the best foundation for developing a uniform and technologically reliable pâté emulsion.

### 3.5. Studying the Chemical Composition of Emulsions

The proximate composition of the eight emulsion variants illustrates how variations in the ratios of by-product mass, vegetable oil, water, and starch affect nutritional balance and technological potential (Table 6). Moisture content ranged from 56.72% (Variant 7) to 66.86% (Variant 3). Formulations with higher water and by-product mass (Variants 3 and 8) showed significantly higher moisture retention compared with the lowest values (*p* < 0.05). This is technologically relevant, as higher moisture improves spreadability and juiciness but can weaken stability if not counterbalanced by protein and starch [37]. Fat content varied strongly, from 19.05% (Variant 3) to 28.44% (Variant 2) (*p* < 0.05). Variants enriched with more vegetable oil (2, 6, and 7) consistently showed higher fat values, supporting creamier texture and energy density. However, excessive fat relative to protein may increase the risk of phase separation. In contrast, Variants 3 and 8 had significantly lower fat, suggesting leaner profiles but with potentially reduced mouthfeel.

Ash values ranged narrowly between 3.16% (Variant 3) and 4.92% (Variant 1), with the largest gap just above 1.7% (*p* < 0.05). Elevated ash in Variants 1, 2, and 5 reflects higher mineral contributions from salts and by-products, which may improve ionic strength and enhance protein solubility. Protein content ranged from 6.62% in Variant 2 to 8.10% in Variant 3, and this variation was statistically significant (*p* < 0.05). Higher protein content in Variants 3 and 8 is attributable to a greater share of by-product mass, improving emulsifying potential. Conversely, Variant 2, with reduced by-product mass and higher oil, had the lowest protein fraction, which may compromise structural stability. Carbohydrate values, reflecting starch addition, ranged from 2.74% (Variant 1) to 7.98% (Variant 4) (*p* < 0.05). As expected, starch-rich formulations (Variants 4 and 7) contained more carbohydrate, which contributes to viscosity, water-holding, and emulsion stability.

The results confirm that formulation balance influences the nutritional and functional properties of emulsions intended for pâtés. Variant 3, with the highest moisture and protein and lowest fat, offers a leaner, protein-focused matrix favorable for emulsification. Variants 2, 6, and 7, with higher fat and moderate moisture, provide richer texture but may require stabilization to prevent fat separation. Variant 4, enriched with starch, shifts toward higher carbohydrate and ash, which enhances viscosity but dilutes protein. Thus, the data emphasize that achieving the optimal pâté emulsion requires balancing fat for palatability, protein for emulsifying stability, and starch for structural reinforcement, with excessive deviation in any component negatively affecting overall quality.

### 3.6. Studying of pH and Water Activity of Emulsion Variants

The pH and water activity values of the eight emulsion formulations reflect the influence of raw material ratios and added stabilizers on the chemical environment and microbial susceptibility of the system. Across all formulations, pH ranged from 7.78 (Variant 6) to 8.15 (Variant 1). The highest pH in Variant 1 (8.15) was significantly greater than in Variants 5 (7.85), 6 (7.78), 7 (7.85), and 8 (7.86) (*p* < 0.05). The observed alkalinity results largely from the addition of baking soda (1%), which raises the buffering capacity of the emulsion. From a technological perspective, slightly alkaline pH can enhance protein solubility and emulsifying potential, supporting stable emulsion formation [38]. However, pH values above 8.0 are unusually high for meat-based systems and may pose concerns regarding microbial ecology and sensory quality. Although short-term processing safety can be maintained through heat treatment and refrigeration, maintaining pH closer to neutrality is generally more desirable for product stability during storage. At higher pH, myofibrillar proteins become more negatively charged. The resulting electrostatic repulsion and swelling can hinder protein crosslinking during heating, leading to weaker gels, a softer pasty texture, and a greater risk of phase separation [39].

Water activity values were uniformly high (0.9923–1.0029), indicating that free water remained abundant in all emulsions. Variants 3 (0.9969), 4 (0.9966), and 8 (1.0029) exhibited significantly higher aw compared to Variants 1 (0.9923) and 6 (0.9926) (*p* < 0.05). High aw facilitates microbial growth potential, reinforcing the need for careful storage conditions.

These findings suggest that alkaline pH improves protein functionality but must be controlled to avoid excessive deviation from typical meat system values. Meanwhile, the consistently high aw indicates that product safety will depend more on thermal processing and cold-chain stability than on intrinsic water limitation.

### 3.7. Studying the Functional–Technological Properties of Emulsions

**Figure 3:**
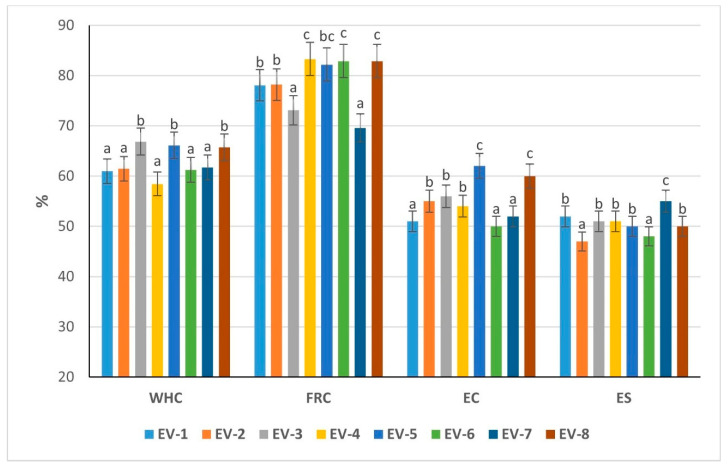
Functional–Technological Properties of Emulsions. Different lowercase letters (a–c) indicate statistically significant differences within the same indicator (*p* < 0.05).

The functional analysis of eight emulsion variants highlighted distinct effects of by-product mass, oil, water, and starch ratios on water-holding (WHC), fat retention (FRC), emulsifying capacity (EC), and emulsion stability (ES) (Figure 3). Variants 3 (66.85%), 5 (66.11%), and 8 (65.76%) showed significantly higher WHC compared with Variants 1 (60.98%), 2 (61.46%), 6 (61.24%), and 7 (61.72%) (*p* < 0.05). This indicates that a higher by-product mass (≥54%) or a more balanced oil–water distribution improves hydration and limits liquid loss during heating, which is an important factor for maintaining pâté yield. Variant 4, with elevated starch (8%), demonstrated the lowest WHC (58.45%), suggesting that excessive starch may disrupt protein hydration.

The highest FRC values were found in Variants 4 (83.3%), 6 (82.9%), 8 (82.9%), and 5 (82.2%), all significantly greater than Variants 1 (78.1%), 2 (78.2%), and especially 7 (69.9%) (*p* < 0.05). These results show that moderate to high starch (4–8%) and sufficient oil incorporation enhance lipid stabilization, whereas lower starch or unfavorable water/oil ratios reduce FRC. Variant 7, despite relatively high oil, exhibited the lowest FRC, indicating insufficient protein–lipid stabilization.

Variant 5 had the highest EC (62%), significantly above Variants 1 (51%), 6 (50%), and 7 (52%) (*p* < 0.05). Variants 3 (56%), 8 (60%), and 2 (55%) also performed well, suggesting that adequate protein availability combined with moderate oil levels enhances interfacial adsorption. Variant 6, despite high FRC, showed the lowest EC, indicating that stability did not originate from high protein activity but likely from starch interactions.

Stability was maximized in Variant 7 (55%), significantly higher than Variants 2 (47%) and 6 (48%) (*p* < 0.05). Variants 3, 4, 5, and 8 (50–51%) demonstrated intermediate stability, while Variant 1 (52%) also maintained acceptable performance. The superior ES of Variant 7 may be linked to its higher starch level (6%), which contributes to viscosity and structural integrity, despite poor FRC.

The results demonstrate that compositional balance is critical for the functional performance of pâté emulsions. High protein (Variants EV3, EV5, EV8) promoted WHC and EC, while starch enrichment (Variants EV4, EV5, EV6, EV8) improved FRC. However, excessive starch (Variant 4) reduced WHC, and excessive oil (Variant EV2) lowered stability. Variant EV5 emerged as a promising formulation, combining high WHC, elevated FRC, and the strongest EC, though with moderate ES. Variant EV7, while less effective in FRC, achieved the highest stability. These findings underline that the optimal emulsion requires balancing protein-driven emulsification with starch-mediated stabilization, ensuring both functional capacity and structural robustness.

### 3.8. Studying of Emulsion Viscosity

**Figure 4:**
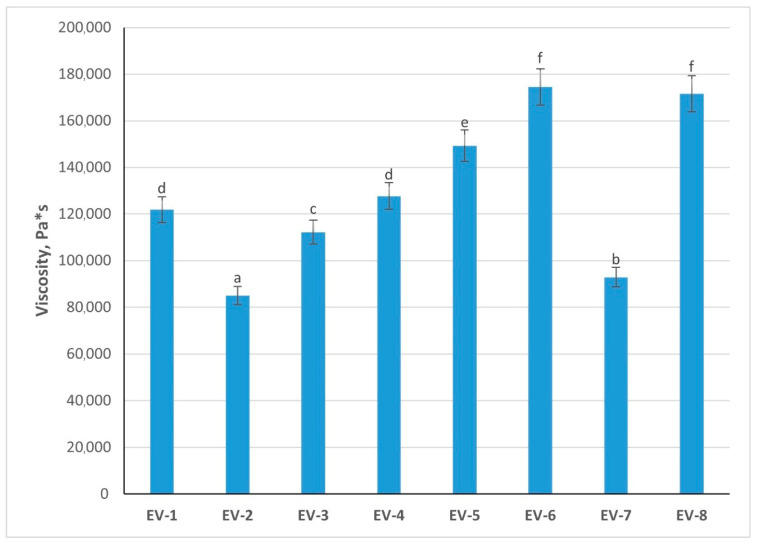
Viscosity of emulsion variants. Different lowercase letters (a–f) indicate statistically significant differences between emulsion variants (*p* < 0.05).

The dynamic viscosity of the eight emulsion variants varied considerably depending on the formulation, reflecting the impact of by-product mass, starch, and oil levels on rheological behavior (Figure 4). Variant EV6 exhibited the highest viscosity (174,481 Pa·s), significantly greater than Variants EV1 (121,825 Pa·s), 2 (85,024 Pa·s), 3 (112,204 Pa·s), and EV7 (92,887 Pa·s) (*p* < 0.05). This suggests that the higher proportion of by-product mass (52%) combined with 22% vegetable oil and 4% starch created a dense protein–starch matrix, resulting in strong resistance to flow. Similarly, Variant EV8 (171,593 Pa·s) and Variant EV5 (149,350 Pa·s) demonstrated elevated viscosity, significantly exceeding lower-viscosity formulations (*p* < 0.05). These results emphasize that a balance of protein concentration and starch incorporation contributes synergistically to thickening, which is favorable for emulsion stability and pâté texture.

Variant EV4 (127,675 Pa·s) showed intermediate viscosity, consistent with its elevated starch level (8%) but lower water content, suggesting starch hydration contributed to structural reinforcement. In contrast, Variants EV2 and EV7 had the lowest viscosities (85,024 and 92,887 Pa·s), significantly lower than most other formulations (*p* < 0.05). These formulations contained higher oil fractions (24–25%) and comparatively lower by-product mass, resulting in weaker protein–starch networking and greater fluidity. Variant EV3 (112,204 Pa·s) was moderate, aligning with its high by-product mass but lower starch level.

Overall, the results demonstrate that viscosity is strongly influenced by protein-to-starch ratio, with starch addition and higher by-product content enhancing structural density. High viscosity, as observed in Variants EV5, EV6, and EV8, is desirable for pâté emulsions, as it correlates with resistance to phase separation and improved textural consistency during processing and storage.

Based on the comparative analysis of the eight emulsion formulations, Variant EV5 was identified as the most suitable for pâté production. This variant demonstrated the strongest balance of functional–technological properties, with significantly higher water-holding (66.11%) and fat retention (82.2%) than Variants EV1, EV2, and EV7 (*p* < 0.05). It also exhibited the highest emulsifying capacity (62%), exceeding Variants EV1, EV6, and EV7 (*p* < 0.05), which is critical for efficient oil incorporation and stable emulsion formation. Variant 5 also maintained a favorable viscosity, providing firmness and resistance to syneresis while retaining spreadability. Its chemical composition corresponds well to pâté quality requirements. Collectively, these results demonstrate that Variant EV5 provides the most reliable foundation for producing a stable, spreadable, and high-quality pâté.

### 3.9. Studying the Chemical Composition of Pâté Variants

The proximate analysis of the five pâté formulations demonstrated measurable changes in nutrient distribution as the proportion of emulsion increased and beef fat was reduced (Table 7). Moisture ranged from 70.65% (Variant PV4) to 71.55% (Variant PV5), confirming that higher emulsion substitution contributed to improved hydration (*p* < 0.05). This effect is consistent with the starch and protein components of the emulsion, which enhance water binding. Protein levels declined progressively from 16.60% in the control (Variant PV1) to 14.54% in Variant PV5. The reduction between Variant PV1 and PV5 (*p* < 0.05) indicates that partial replacement of poultry meat and liver with emulsion lowers the overall protein fraction. This has functional implications: while protein content is crucial for emulsifying capacity, the decline was moderate and offset by improved stability indices noted in earlier tests.

Fat content ranged from 7.39% in Variant PV5 to 8.51% in Variant PV4, and this difference was statistically significant (*p* < 0.05). The higher fat level in Variant 4 reflects the balance of residual beef fat and emulsion lipids, while Variant PV5, prepared without beef fat, contained less lipid. This supports the nutritional objective of reducing saturated animal fat in pâté while maintaining acceptable sensory texture. Ash content increased from 2.02% (Variant PV1) to 2.86% (Variant PV5), with the rise between these extremes statistically significant (*p* < 0.05). This reflects the contribution of added salt and mineral content from the emulsion. Carbohydrate levels, largely supplied by starch in the emulsion, rose significantly from 2.76% in Variant PV1 to 3.66% in Variant PV5 (*p* < 0.05). This shift is important technologically, as starch contributes to viscosity, water retention, and emulsion stability.

The data confirm that increasing emulsion substitution leads to a gradual reduction in protein and fat, while enhancing ash and carbohydrate fractions. Technologically, this shift supports better water binding and emulsion stability. Nutritionally, reducing animal fat and slightly raising carbohydrate content helps create pâtés with lower saturated fat while maintaining good product quality, provided protein levels remain sufficient to sustain emulsifying capacity. Collectively, Variant 5 demonstrated the most pronounced compositional shift, providing evidence that the developed emulsion is an effective functional fat replacer in pâté systems.

### 3.10. Studying Functional–Technological Properties of Pâté Variants

**Figure 5:**
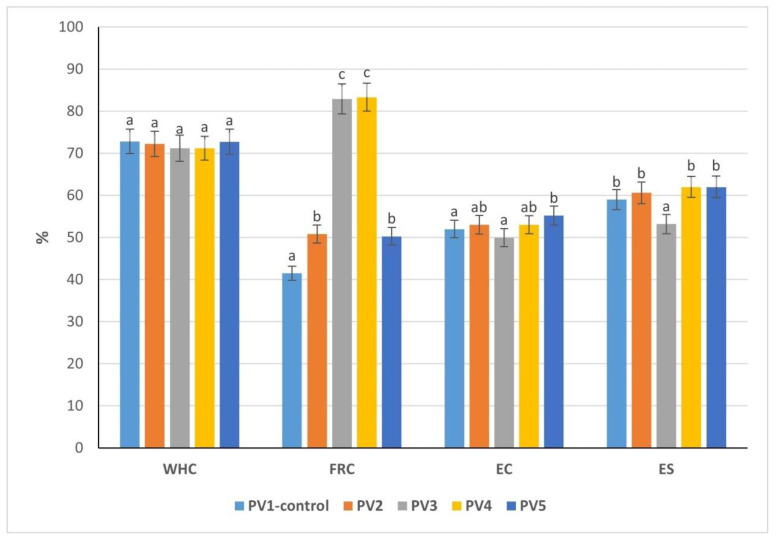
Functional–Technological Properties of Pâté Variants. Different lowercase letters (a–c) indicate statistically significant differences within the same indicator (*p* < 0.05).

The incorporation of emulsion significantly influenced the functional indicators of the pâté formulations compared with the control variant (Figure 5). For WHC values ranged from 71.2% (Variants PV3 and PV4) to 72.85% (Variant PV1). The differences between Variant PV1 (72.85%) and Variants PV3–4 (71.2%) were statistically significant (*p* < 0.05), suggesting that increasing emulsion substitution reduced the ability of the system to bind water. However, Variant PV5 (72.73%) restored WHC close to control levels, indicating that at higher emulsion levels (25%), starch and protein interactions compensated for water loss.

Marked differences were observed on FRC values, ranging from 41.5% (Variant PV1) to 83.3% (Variant PV4). The increase in FRC in Variants PV3 and PV4 (82.9% and 83.3%) was statistically significant compared with the control (*p* < 0.05), demonstrating that emulsion addition effectively stabilizes fat, minimizing losses during heating. Variant PV5 showed lower FRC (50.3%) despite high emulsion content, likely due to the absence of beef fat and altered fat distribution.

EC values were relatively stable (50–55.2%), with the highest value in Variant PV5 (55.2%), significantly greater than Variant PV3 (50%) (*p* < 0.05). This suggests that the structural proteins in the emulsion provided additional interfacial activity when beef fat was fully replaced.

ES ranged from 53.2% (Variant PV3) to 62% (Variants PV4 and PV5). Variant PV4 achieved significantly higher stability than Variant 3 (*p* < 0.05), highlighting that increasing the emulsion to 18.75% improves the resistance to phase separation. Variant PV5 maintained similarly high stability, confirming the effectiveness of the emulsion in reinforcing the structure.

These results demonstrate that replacing beef fat with protein–fat emulsion enhances fat retention and emulsion stability, critical for pâté quality. Variants PV3 and PV4 provided the best FRC, while Variant PV5 balanced improved emulsifying capacity and ES despite slightly reduced fat retention. Collectively, emulsion incorporation allowed for reduced animal fat without compromising technological performance, supporting the research objective of producing healthier pâtés with stable structure and acceptable functional properties.

### 3.11. Determination of Yield Stress of Pâté Variants

**Figure 6:**
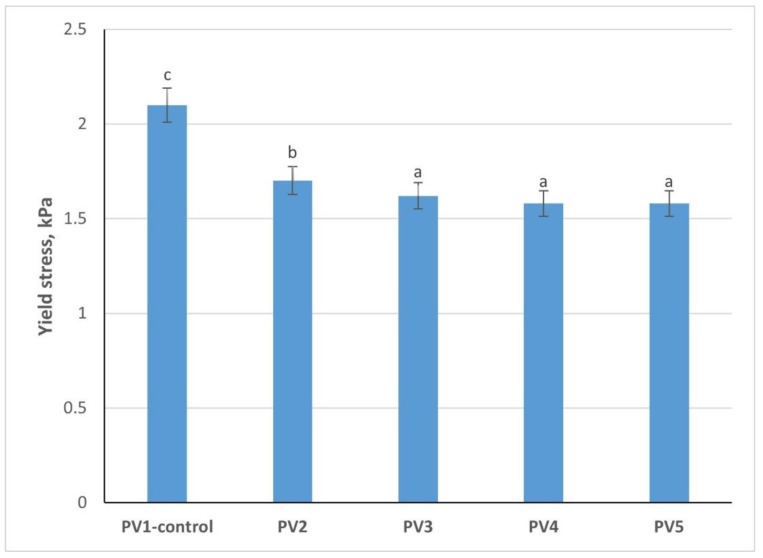
Yield stress of Pâté Variants. Different lowercase letters (a–c) indicate statistically significant differences between pâté variants (*p* < 0.05).

The measurement of yield stress provides insight into the structural strength of the pâté mixtures, indicating the shear force required to initiate flow and thus reflecting texture and spreadability (Figure 6). Variant PV1 (without emulsion) exhibited the highest yield stress (2.1 kPa), significantly higher than all emulsion-containing samples (*p* < 0.05). This indicates a firmer and denser matrix in the traditional formulation, largely attributable to the presence of beef fat and higher levels of intact muscle proteins. Incorporation of emulsion consistently reduced yield stress. Variant PV2 dropped to 1.7 kPa, while Variants 3–5 showed further reductions (1.62–1.58 kPa). The decline of approximately 25% compared with the control (*p* < 0.05) suggests that partial replacement of animal fat with emulsion softens the structure, making the product more spreadable. Among the emulsion-containing formulations, differences between Variants PV3, 4, and 5 were not statistically significant, indicating that beyond 12.5% emulsion substitution, the yield stress stabilizes.

Reduced yield stress is not inherently negative. While Variant PV1 offered the most compact structure, its firmness may compromise consumer preference for pâté, which is typically expected to be smooth and easily spreadable. Variants 3–5, with lower yield stress, more closely align with desired textural characteristics, balancing structural integrity with spreadability. These results confirm that replacing beef fat with protein–fat emulsion decreases yield stress, leading to softer pâtés. Variants PV3–5 provide the most favorable rheological properties for consumer-acceptable spreadability without loss of structural stability.

### 3.12. Studying of pH and Water Activity of Pâté Variants

**Figure 7:**
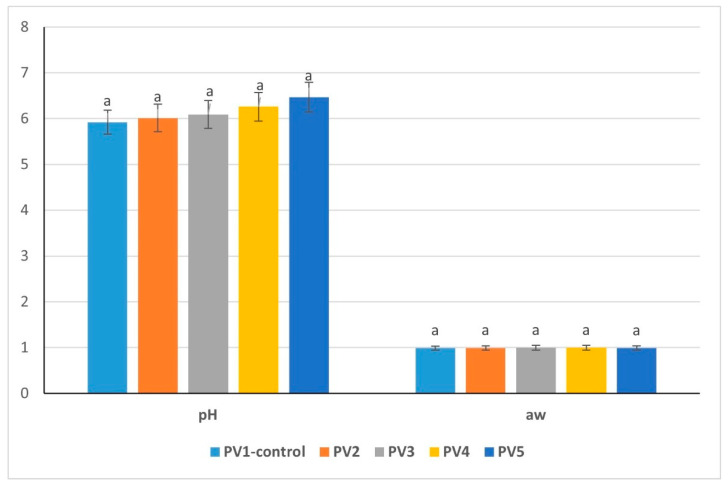
pH and Water Activity of Pâté Variants. Same lowercase letters (a) indicate no statistically significant differences within the same indicator (*p* > 0.05).

The pH of the pâté masses ranged from 5.92 in the control (Variant PV1) to 6.47 in Variant PV5. The increase was progressive with higher levels of emulsion incorporation. Variants 4 (6.26) and 5 (6.47) were significantly higher compared with Variants PV1 and PV2 (*p* < 0.05) (Figure 7). This shift can be attributed to the alkaline contribution of the emulsion ingredients, particularly sodium bicarbonate, which elevates pH. From a technological perspective, higher pH enhances protein solubility and water-holding capacity, which may support emulsion stability. However, excessive alkalinity may alter flavor and affect microbial ecology.

Water activity values were uniformly high, ranging from 0.9885 in Variant 5 to 0.9962 in Variant PV3 (*p* < 0.05). Variant PV3, with intermediate emulsion content, displayed the highest aw, suggesting increased availability of free water, which may increase microbial susceptibility.

The combination of higher pH and consistently high water activity confirms that pâté emulsions require careful control of heat treatment and storage conditions to ensure microbiological safety. Moderate increases in pH improve protein functionality, but values above 6.4, as in Variant 5, should be closely monitored for sensory acceptability and stability during storage. Collectively, these results highlight the dual role of emulsion addition: improving functional properties through pH elevation, while simultaneously influencing water availability and microbial risk.

### 3.13. Studying the Microstructure of Pâté Variants

**Figure 8:**
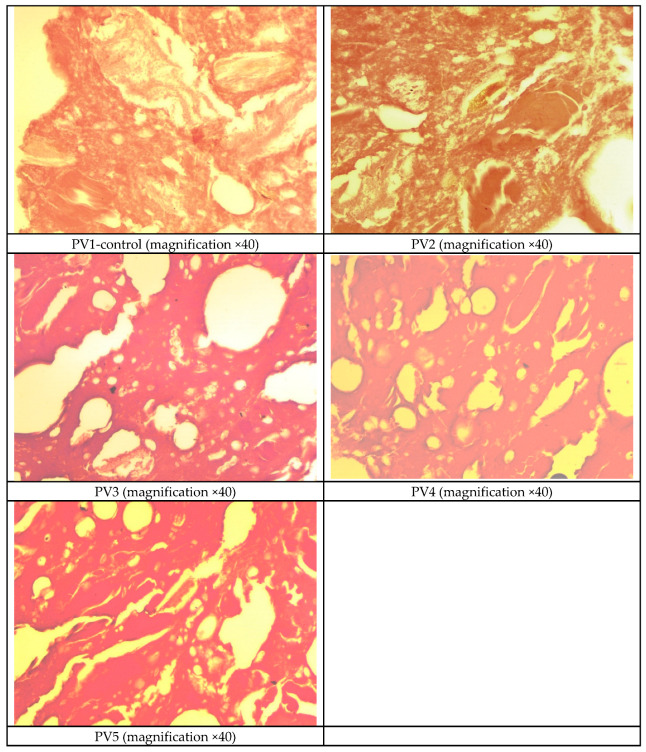
Microstructure analysis of pate variants.

The microstructure of pâté provides critical insights into how proteins, lipids, and water interact within the matrix, directly influencing emulsion stability, texture, juiciness, and yield. Microscopic examination allows identification of fat droplet distribution, protein network formation, and void characteristics, which together determine the technological performance of each formulation. The following observations (Figure 8) describe structural differences among the studied variants and link them to functional outcomes.

The microstructure of PV1-control (without emulsion) shows a dense and compact protein network formed from coagulated poultry meat and liver proteins, with scattered oval voids corresponding to fat droplets. These droplets appear poorly dispersed and weakly stabilized due to the absence of added protein–fat emulsion. As a result, lipid distribution within the matrix is uneven, and interfacial coverage between protein and fat phases is limited. Such a structure indicates lower emulsifying stability and weaker water- and fat-binding, which can lead to higher cooking losses, reduced juiciness, and a firmer, less spreadable texture compared to emulsion-containing variants.

The microstructure of PV2 shows a more heterogeneous and open network compared to Variant 1. The image reveals bundles of muscle fibers that are loosely arranged and interconnected, forming a visible fibrous framework. Numerous small round and oval voids, appearing as white areas, are dispersed throughout the matrix and are more frequent than in Variant PV1, suggesting a higher presence of fat globules or air pockets. These voids are evenly distributed within the protein–lipid mass, contributing to the overall porosity of the product. The muscle fiber networks provide structural cohesion, while the higher frequency of voids indicates less dense packing of the matrix. This combination suggests that Variant PV2 has a more open structure with greater porosity, which may influence its textural softness and water or fat retention capacity compared to the denser and more compact Variant 1.

The microstructure of PV3 shows a distinctly heterogeneous fat distribution. Large, round and oval voids are clearly visible, with several oversized fat droplets suggesting localized accumulation rather than uniform dispersion. These large voids create wide separations between muscle fiber regions, reducing the compactness of the protein–fat matrix. The surrounding muscle fibers appear monolithic and homogeneous, but the presence of narrow channels and gaps highlights an imbalance in emulsion stabilization. This morphology indicates partial destabilization, where protein films were insufficient to maintain fine droplet dispersion, leading to fat coalescence and reduced structural uniformity compared to more stable variants.

The microstructure of PV4 reveals a mixed protein–fat matrix with numerous round and oval voids. Several of these voids are large, suggesting localized accumulation of fat and indicating an uneven distribution of fat globules. Muscle fibers appear dense, compact, and relatively homogeneous, with occasional narrow voids present. This structure points to incomplete stabilization of fat droplets within the protein network, which may influence emulsion stability and fat retention.

The microstructure of PV5 presents a homogeneously mixed matrix in which muscle fibers appear compact and monolithic. Numerous round and oval voids are visible, representing fat droplets dispersed within the protein network. Compared to other variants, the voids are smaller and more evenly distributed, suggesting improved integration of fat into the matrix. Narrow channels and voids are also present, but they do not dominate the structure, indicating that the protein network maintains cohesion and structural integrity. This variant demonstrates a relatively stable emulsion with balanced distribution of aqueous and lipid phases, supporting desirable textural and functional properties.

Overall, the microstructural analysis demonstrates that the addition and composition of protein-fat emulsions strongly influence droplet distribution, protein network cohesion, and void formation, with emulsion-containing variants showing more uniform matrices and greater structural stability compared to the control without emulsion.

### 3.14. Studying Drip Loss in Pâté Variants

Drip loss after cooking is a critical indicator of pâté quality, as it reflects the extent to which the protein–fat–water matrix can retain bound components during heat treatment. Excessive broth release signals poor binding and negatively affects texture, juiciness, and yield [40,41]. Drip loss increases when the cooked protein network is weak. Causes include poor extraction of myofibrillar proteins (too little salt, pH near the isoelectric point), batter overheating, inadequate starch gelatinization, and unstable fat droplets. Low shear, long holding before cooking, and an imbalanced fat-to-protein ratio further worsen separation [42].

Variant PV1 (without emulsion) showed a drip loss of 9.24%, which can be considered the baseline for comparison. Variant PV2 had the highest drip loss (11.35%), significantly greater than Variant PV1 (*p* < 0.05). This suggests that partial replacement of meat and fat with emulsion at 6.25% was insufficient to stabilize the protein matrix, leading to weaker binding of moisture and fat. In contrast, Variant PV3 displayed the lowest loss among intermediate levels (7.95%), significantly lower than Variant PV2 (*p* < 0.05), indicating that increasing emulsion to 12.5% improved structural integrity and reduced purge. Variant 4 (8.71%) showed intermediate performance, not differing significantly from the control (Table 8).

Variant PV5 (25% emulsion) exhibited the lowest drip loss overall (3.6%), significantly lower than all other formulations (*p* < 0.05). This indicates that a higher proportion of emulsion enhances water and fat binding during thermal treatment, likely due to improved protein solubility, starch gelation, and the formation of a stable protein–polysaccharide–lipid network.

The results confirm that the inclusion of emulsion can markedly improve cooking stability in pâtés by reducing drip loss. While low substitution levels (Variant PV2) may destabilize the system, higher additions (Variants PV3–5) progressively enhance retention, with Variant PV5 demonstrating the most effective binding. These findings highlight the technological value of emulsion in replacing animal fat while simultaneously improving yield and product quality.

### 3.15. Studying of Organoleptic Properties of Pâté Variants

**Figure 9:**
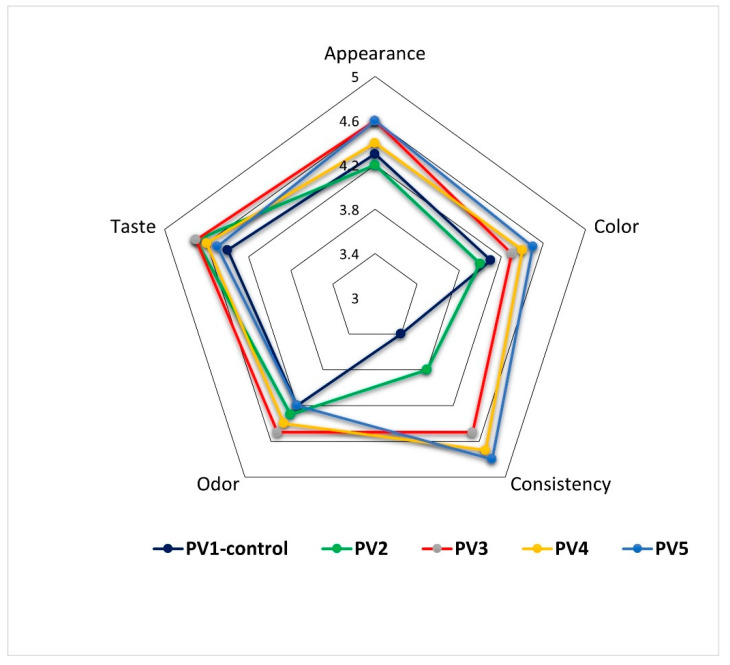
Organoleptic Score of Pâté Variants.

Organoleptic evaluation demonstrated that the incorporation of emulsion positively influenced sensory attributes of the pâtés. The control sample (Variant PV1) received the lowest total score (20.4 points), while Variants PV3–5 achieved the highest scores (22.6 points each). Improvements were particularly evident in consistency and appearance, which are critical determinants of consumer acceptance (Figure 9).

Appearance scores ranged between 4.2 and 4.6, with Variant PV3 and PV5 receiving significantly higher scores than Variant PV2 (*p* < 0.05). Color was also improved in emulsion-rich samples, with Variant PV5 reaching 4.5 compared with 4.0 in Variant PV2 (*p* < 0.05). The increased brightness and uniformity are likely attributable to starch and protein in the emulsion, which enhance gel formation and surface smoothness. Consistency showed the most pronounced differences, rising from 3.4 in the control (Variant PV1) to 4.8 in Variant PV5. The difference between these two formulations is statistically significant (*p* < 0.05). This demonstrates that partial or full replacement of beef fat with emulsion improves spreadability and texture homogeneity, a key objective of the study. Odor scores remained relatively stable (4.2–4.5), with no significant differences between variants. Taste scores were consistently high (4.4–4.7), with Variant PV2 and Variants PV3–5 slightly exceeding the control, though differences were not statistically significant. This indicates that emulsion substitution did not negatively impact flavor perception.

The sensory data confirm that emulsion incorporation enhances organoleptic quality, particularly texture, without compromising taste or odor. Variants PV3–5 represent the most consumer-acceptable formulations, with Variant 5 achieving the best balance of appearance, color, and consistency. Organoleptic evaluation demonstrated that emulsion-rich pâtés are not only technologically superior but also more appealing to consumers.

### 3.16. Determination of Total Viable Count in Pâté Variants

The total viable count (TVC) of the five pâté variants was examined both before and after heat treatment. Before sterilization, TVC values ranged between 930 CFU/g in Variant 1 and 830 CFU/g in Variant PV5, while after sterilization, no viable microorganisms were detected in any sample. This demonstrates that the applied thermal process was highly effective, ensuring microbial safety across all formulations.

Pre-cooking microbiological profile.

The gradual reduction in TVC from Variant PV1 to PV5 indicates a small but consistent effect of emulsion incorporation on the microbial load (Table 9). Variant PV1, the control without emulsion, showed the highest microbial count (930 CFU/g), whereas Variant PV5, with the highest level of emulsion, showed the lowest count (830 CFU/g). The differences between Variant PV1 and Variants PV4-5 were statistically significant (*p* < 0.05). This trend can be explained by the presence of salt, starch, and bicarbonate in the emulsion, which provide mild bacteriostatic effects and may reduce the overall microbial contribution from raw fat sources.

Post-cooking microbial safety.

After sterilization, TVC values were not detected in any of the variants. This outcome confirms the adequacy of the heat treatment in destroying vegetative microorganisms and spores, thus ensuring product stability and compliance with microbiological safety requirements. Importantly, the elimination of microorganisms after processing indicates that small pre-cooking differences between variants do not compromise the final safety of the product.

These results demonstrate that replacing animal fat with protein–fat emulsions does not increase the microbial load of pâtés and, in fact, can slightly reduce initial counts. More importantly, the sterilization step completely eliminates microbial risks, confirming that pâtés formulated with chicken by-product emulsions are microbiologically safe. This reinforces the technological feasibility of using such emulsions in pâté production while ensuring compliance with food safety standards.

### 3.17. Studying TBARS Values in Pâté Variants During Storage

**Figure 10:**
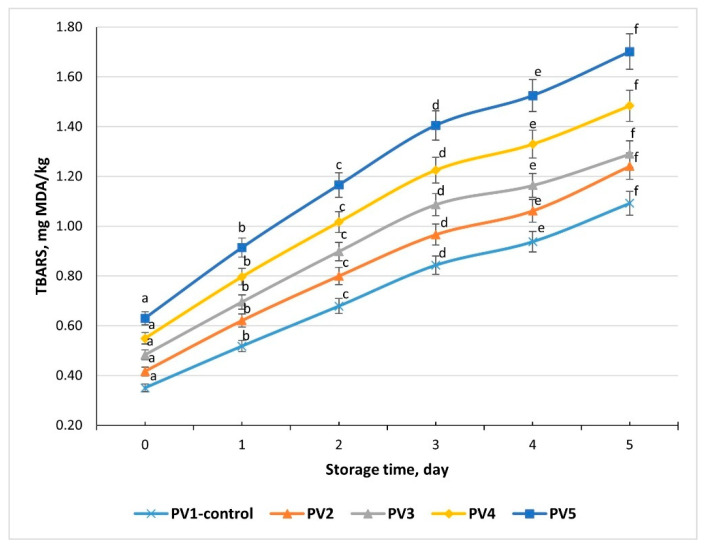
TBARS Values in Pâté Variants During Storage. Different lowercase letters (a–f) indicate statistically significant differences within the storage time of pâté variants (*p* < 0.05).

Across all five pâté formulations, TBARS values increased progressively during refrigerated storage (+2 to +4 °C) from day 0 to day 5, confirming the development of lipid oxidation in cooked meat systems. The control sample (Variant PV1) rose from 0.35 to 1.09 mg MDA/kg, while Variant PV5, with the highest level of emulsion substitution, increased from 0.63 to 1.70 mg MDA/kg (Figure 10). The smooth increase without irregular fluctuations indicates that the analytical method was consistent and reliable.

At day 0, TBARS values were significantly higher in Variant 5 compared with Variant 1 (*p* < 0.05), reflecting the greater proportion of vegetable oil and its higher susceptibility to oxidation. This pattern persisted throughout storage: after 5 days, TBARS in Variant PV5 (1.70) remained significantly greater than Variants PV1 (1.09) and 2 (1.24) (*p* < 0.05). Variants PV3 and 4 showed intermediate values, confirming that the extent of emulsion substitution strongly influenced oxidative stability.

The increase in TBARS with rising emulsion levels can be attributed to the higher proportion of polyunsaturated fatty acids (PUFA) from vegetable oil, which oxidize more rapidly than saturated fats from beef tallow [43]. Furthermore, emulsified systems present a larger interfacial surface area, facilitating oxygen diffusion and free radical propagation. Elevated pH in higher-emulsion variants may also accelerate oxidation [44].

The data demonstrate that while emulsion incorporation improves functional properties and reduces animal fat, it also increases susceptibility to lipid oxidation. TBARS analysis confirms that lipid oxidation progressed steadily during storage, with the rate and extent significantly higher in emulsion-rich variants (*p* < 0.05). Thus, although Variant 5 shows the best functional performance, its higher oxidative instability must be addressed to ensure product quality and shelf life.

### 3.18. Studying Acid Number in Pâté Variants During Storage

**Figure 11:**
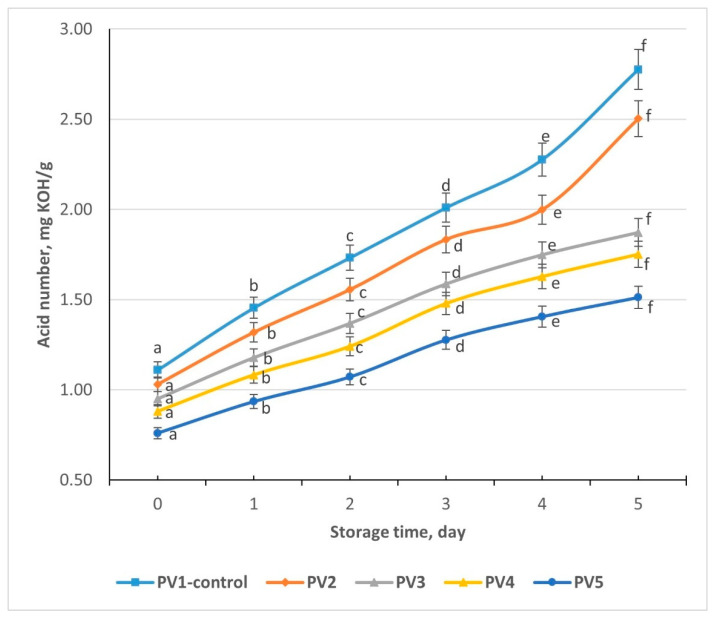
Acid Number in Pâté Variants During Storage. Different lowercase letters (a–f) indicate statistically significant differences within the storage time of pâté variants (*p* < 0.05).

Across all five pâté formulations, the acid number increased progressively over 5 days of refrigerated storage at +2 to +4 °C. This indicates hydrolytic changes in the lipid fraction, driven by residual lipases/phospholipases and possible secondary oxidation products [45]. The control (Variant PV1) increased from 1.11 mg KOH/g at day 0 to 2.78 mg KOH/g at day 5, while Variant PV5, containing the highest proportion of emulsion and no beef fat, rose from 0.76 to 1.51 mg KOH/g (Figure 11).

At day 0, acid number values were significantly lower in Variant 5 compared with Variant PV1 (difference > 2.9%, *p* < 0.05). This reflects the substitution of animal fat with refined vegetable oil, which inherently has a lower free fatty acid content. This pattern was consistent throughout storage: by day 5, Variant PV5 (1.51 mg KOH/g) remained significantly lower than Variants PV1 (2.78) and PV2 (2.50) (*p* < 0.05). Variants PV3 and PV4 showed intermediate values, supporting the direct influence of emulsion level on AN progression.

The steady rise in acid number across all samples is typical of meat products, especially those containing liver, which provides phospholipids and lipase activity. The higher moisture and near-neutral pH of the pâté matrix facilitate hydrolytic reactions. These results demonstrate that partial replacement of animal fat with emulsion effectively lowers the acid number at production and slows its increase during storage, thereby reducing hydrolytic rancidity. This supports the hypothesis that emulsion-based formulations not only reduce animal fat but also improve lipid stability. Acid number analysis confirms that emulsion-rich variants (particularly Variant PV5) are less prone to hydrolytic rancidity than the control, representing a significant technological advantage in pâté formulation.

## 4. Discussion

Emulsions in comminuted meats act as structural systems that control water and fat retention, cooking yield, texture, and oxidative stability. These functions depend on interfacial film formation, droplet size, viscosity of the continuous phase, and pH. As a result, the emulsion’s composition directly impacts the quality of the final product. Choi et al. (2009) reported that replacing pork backfat with pre-emulsified vegetable oils plus rice-bran fiber increased batter viscosity, moisture and protein, raised pH, and reduced cooking loss while improving emulsion stability and gel strength [46]. These results are comparative to our study, which also shows higher viscosity and stability when the matrix is reinforced. Chen et al. (2015) observed that soybean oil pre-emulsified with chicken plasma protein produced lower cooking and press losses, higher apparent viscosity, and more homogeneous fat distribution than unemulsified oil; textural cohesion increased without excessive hardness [47]. This study yielded similar results, showing that structured oil phases similarly increased stability and retention. Bañón et al. (2008) demonstrated that higher emulsification temperature predicts greater cooking loss and softer gels, whereas added starch and functional protein strengthen the network [48]. These findings align with the present data in which fine comminution and starch gelatinization supported water and fat binding. Clark (2013) [49] and Li et al. (2020) [50] reported that smaller droplets and higher continuous-phase viscosity improve emulsion stability via interfacial design. The present results are consistent with this mechanism and yielded stable dispersions. Bajčić et al. (2023) showed that inulin–collagen systems can replace animal fat while maintaining technological quality, consistent with the role of biopolymers observed here [51]. Pereira et al. (2020) found that pre-emulsified sunflower oil with plant co-ingredients reduced cooking loss and increased emulsion stability [52], which parallels the stability improvements recorded in the present emulsions. Okuskhanova et al. (2023) reported that caseinate–rumen–sunflower emulsions improved water and fat holding and shifted the fatty-acid profile toward higher PUFA [53]. These results are comparable to the present study, which demonstrates that structured interfaces raise retention metrics.

Collectively, the literature indicates that interfacial engineering, adequate shear, temperature control, and polysaccharide thickening elevate viscosity, water and fat holding, and emulsion stability. These results are comparable to our study, in which a by-product protein emulsion with starch and controlled alkalinity achieved similar functional gains while enabling valorization of poultry side-streams.

These emulsion principles also apply directly to finished products such as pâtés. Accordingly, pâté reformulations increasingly use structured or liquid vegetable oils to preserve technological performance while improving the lipid profile. In chicken liver pâtés with refined sunflower oil, acceptable composition, pH, and storage stability were maintained, and a formulation with twenty-eight percent oil was favored on nutritional and quality properties [54]. When plant oils are pre-emulsified before incorporation, cooking loss tends to decrease and sensory spreadability improves, provided batter viscosity is sufficient to stabilize droplets [55,56]. Reformulating the lipid fraction with health-oriented oils elevates polyunsaturated fatty acids and can increase susceptibility to oxidation unless antioxidant strategies are used [57,58].

The present pâtés, formulated with a protein–fat emulsion, exhibited physicochemical values consistent with these trends. Post-cook pH ranged from 6.07 to 6.34 and water activity from 0.988 to 0.993. Water retention and fat retention after cooking were high, and emulsion stability was 51 to 63 percent, indicating effective immobilization of aqueous and lipid phases. Cooking losses were controlled, as reflected by low drip after heating, and sensory evaluation favored emulsion-containing variants, paralleling reports that oil-reformulated pâtés are perceived as more spreadable and acceptable [54,55].

Oxidative and hydrolytic indices showed the typical changes that occur with different formulations. TBARS increased over five days of refrigeration and were higher at greater emulsion inclusion. This pattern matches reports for pâtés enriched with polyunsaturated oils, while formulations paired with antioxidants showed restrained or delayed TBARS increases [56,57,58]. In contrast, acid value at production was lower in emulsion-rich variants and rose more slowly during storage, indicating reduced hydrolytic rancidity relative to the control, which aligns with the use of refined oil within a stabilized matrix.

To summarize, the results confirm that emulsion-based reformulation supports both nutritional and technological improvements in pâtés. Functional stability was maintained through effective water and fat retention, while sensory acceptance was enhanced by a softer, spreadable texture. At the same time, oxidative stability emerged as the main limitation, reinforcing the need for antioxidant strategies when polyunsaturated oils are included. Overall, the findings demonstrate that poultry by-product emulsions can deliver functional gains while reducing reliance on conventional animal fat.

Compared with commercial pâtés (for example, Kubley (Kazakhstan), Glavprodukt (Russia), Ruzkom (Russia), and Hame (Czech Republic) available in Kazakhstan’s market, the developed pâté with protein–fat emulsion contained markedly less fat (7.39% vs. 20–30%) and higher moisture (71.55%) and ash (2.86%) contents. Protein (14.54%) remained within or above the typical range (8–13%), while carbohydrates increased to 3.66% due to starch in the emulsion. The replacement of animal fat with vegetable oil enriched in polyunsaturated fatty acids significantly reduced the amount of saturated lipids, yielding a nutritionally leaner and more functionally stable product. These compositional shifts confirm the emulsion’s effectiveness as a fat replacer, enhancing quality and health value.

## 5. Conclusions

This study demonstrated that chicken by-products (hearts, gizzards, and skin) can be effectively utilized as raw materials for protein–fat emulsions to replace animal fat in pâté formulations. The proximate composition of the by-products influenced emulsion functionality, with protein-rich fractions enhancing emulsifying capacity and skin-derived lipids supporting texture. Emulsion composition and balance of protein, starch, and oil determined technological performance, affecting water-holding, fat retention, stability, and viscosity. In pâté systems, emulsion addition modified chemical composition by lowering protein and fat while increasing ash and carbohydrate levels, contributing to improved hydration and functional stability. Technological evaluation confirmed that higher emulsion levels reduced drip loss, enhanced spreadability, and improved sensory quality without compromising microbial safety. Although lipid oxidation increased with greater inclusion of vegetable oil, hydrolytic rancidity was lower in emulsion-rich variants, indicating improved lipid stability. Among the tested formulations, Variant PV5 with the highest emulsion content achieved the most favorable balance of physicochemical, functional, and sensory properties. These findings confirm the technological feasibility of using chicken by-product emulsions as fat replacers in pâtés, offering a sustainable approach to valorize poultry side-streams while producing healthier, high-quality meat products.

## Figures and Tables

**Table 1:** Formulations of different variants of chicken by-product mixture.

Name of By-Product	Mass Fraction, %			
	BMV-1	BMV-2	BMV-3	BMV-4
Chicken skin	34	50	25	25
Gizzards	33	25	50	25
Hearts	33	25	25	50
Total	100	100	100	100

BMV—by-product mixture variant.

**Table 2:** Formulations of different variants of emulsions.

Emulsion’s Variant	Chicken By-Product Mixture	Sunflower Oil	Water	Potato Starch	Sodium Bicarbonate	Sodium Chloride
EV-1	50	20	25	3	1	1
EV-2	45	25	25	3	1	1
EV-3	55	15	25	3	1	1
EV-4	50	20	20	8	1	1
EV-5	48	18	28	4	1	1
EV-6	52	22	20	4	1	1
EV-7	46	24	22	6	1	1
EV-8	54	16	24	4	1	1

EV—emulsion’s variant.

**Table 3:** Formulation of different variants of pates.

Ingredient	PV1-Control	PV2	PV3	PV4	PV5
Chicken meat	60.7	55.9	51.1	46.3	41
Chicken liver	17.6	17.6	17.6	17.6	17.6
Beef fat	5.3	4.3	3.3	2.3	0
Emulsion	0	6.25	12.5	18.75	25
Onion	6.3	6.3	6.3	6.3	6.3
Carrot	5.7	5.7	5.7	5.7	5.7
Parsley root	0.6	0.6	0.6	0.6	0.6
Ground black pepper	0.05	0.05	0.05	0.05	0.05
Salt	1.05	1.05	1.05	1.05	1.05
Broth	2.7	2.25	1.8	1.35	2.7

PV—pate’s variant.

**Table 4:** Chemical composition of chicken by-products.

Chicken By-Product	Moisture	Protein	Fat	Ash
Heart	75.88 ± 0.79 ^b^	16.30 ± 0.16 ^b^	6.22 ± 0.12 ^c^	1.60 ± 0.02 ^c^
Liver	77.73 ± 0.55 ^b^	19.10 ± 0.30 ^c^	1.24 ± 0.02 ^a^	1.93 ± 0.03 ^d^
Gizzard	75.54 ± 1.27 ^b^	18.59 ± 0.32 ^c^	4.57 ± 0.09 ^b^	1.30 ± 0.02 ^b^
Skin	59.72 ± 0.82 ^a^	15.06 ± 0.23 ^a^	24.46 ± 0.43 ^d^	0.76 ± 0.01 ^a^

^a–d^*p* < 0.05.

**Table 5:** Chemical composition of chicken by-product mixture.

Indicator	BMV1	BMV2	BMV3	BMV4
Protein	17.90 ± 0.23 ^c^	15.51 ± 0.18 ^b^	16.07 ± 0.21 ^b^	14.71 ± 0.20 ^a^
Fat	11.88 ± 0.21 ^b^	14.93 ± 0.15 ^c^	9.96 ± 0.07 ^a^	10.37 ± 0.10 ^a^
Ash	2.51 ± 0.04 ^b^	2.45 ± 0.02 ^b^	2.04 ± 0.04 ^a^	2.10 ± 0.03 ^a^
Moisture	67.71 ± 1.37 ^a^	67.11 ± 1.11 ^a^	71.94 ± 1.19 ^b^	72.82 ± 1.39 ^b^

^a–c^*p *< 0.05. BMV—by-product mixture variant.

**Table 6:** Chemical Composition of Emulsions.

Emulsion’s Variant	Moisture, %	Fat, %	Ash, %	Protein, %	Carbohydrates, %
EV-1	61.14 ± 1.00 ^b^	23.84 ± 0.40 ^d^	4.92 ± 0.08 ^d^	7.36 ± 0.14 ^cd^	2.74 ± 0.05 ^a^
EV-2	57.77 ± 0.88 ^a^	28.44 ± 0.45 ^f^	4.36 ± 0.09 ^c^	6.62 ± 0.08 ^a^	2.81 ± 0.05 ^a^
EV-3	66.86 ± 1.35 ^c^	19.05 ± 0.25 ^a^	3.16 ± 0.05 ^a^	8.10 ± 0.15 ^e^	2.83 ± 0.06 ^a^
EV-4	57.14 ± 0.63 ^a^	23.82 ± 0.45 ^d^	3.43 ± 0.05 ^b^	7.63 ± 0.13 ^d^	7.98 ± 0.16 ^e^
EV-5	62.95 ± 0.72 ^b^	21.84 ± 0.38 ^c^	4.16 ± 0.07 ^c^	7.06 ± 0.10 ^b^	3.99 ± 0.06 ^c^
EV-6	58.87 ± 1.05 ^a^	26.71 ± 0.58 ^e^	3.20 ± 0.03 ^a^	7.65 ± 0.12 ^d^	3.57 ± 0.05 ^b^
EV-7	56.72 ± 0.87 ^a^	26.94 ± 0.38 ^e^	3.59 ± 0.08 ^b^	6.77 ± 0.13 ^a^	5.98 ± 0.13 ^d^
EV-8	64.01 ± 1.12 ^bc^	20.61 ± 0.21 ^b^	3.60 ± 0.05 ^b^	7.94 ± 0.13 ^d^	3.84 ± 0.07 ^c^

^a–f^ Different lowercase letters indicate statistically significant differences within the columns (*p* < 0.05). EV—Emulsion variant.

**Table 7:** Chemical composition of pate variants.

Indicator	PV1-control	PV2	PV3	PV4	PV5
Moisture	70.90 ± 1.09 ^a^	70.92 ± 0.99 ^a^	70.74 ± 1.15 ^a^	70.65 ± 1.01 ^a^	71.55 ± 1.16 ^a^
Protein	16.60 ± 0.23 ^c^	16.01 ± 0.17 ^bc^	15.41 ± 0.28 ^b^	14.76 ± 0.19 ^a^	14.54 ± 0.15 ^a^
Fat	7.72 ± 0.10 ^ab^	7.97 ± 0.11 ^b^	8.21 ± 0.09 ^bc^	8.51 ± 0.06 ^c^	7.39 ± 0.11 ^a^
Ash	2.02 ± 0.03 ^a^	2.11 ± 0.04 ^a^	2.43 ± 0.04 ^b^	2.78 ± 0.03 ^c^	2.86 ± 0.05 ^c^
Carbohydrates	2.76 ± 0.04 ^a^	2.99 ± 0.03 ^b^	3.21 ± 0.05 ^c^	3.30 ± 0.04 ^c^	3.66 ± 0.05 ^d^

^a–d^ Different lowercase letters indicate statistically significant differences within the rows (*p* < 0.05). PV—pate variant.

**Table 8:** Drip loss in pâté variants after heat treatment.

Pate’s Variant	Drip loss (%)
PV1-control	9.24 ± 0.11 ^d^
PV2	11.35 ± 0.19 ^e^
PV3	7.95 ± 0.15 ^b^
PV4	8.71 ± 0.14 ^c^
PV5	3.60 ± 0.06 ^a^

^a–e^ Different lowercase letters indicate statistically significant differences (*p* < 0.05).

**Table 9:** Total viable count in pâté variants.

Indicator	PV1-Control	PV2	PV3	PV4	PV5
TVC before cooking	930	904	880	855	830
TVC after cooking	nd	nd	nd	nd	nd

## Data Availability

The original contributions presented in the study are included in the article/Supplementary Materials; further inquiries can be directed towards the corresponding author.

## Supplementary Materials

The following supporting information can be downloaded at: https://www.mdpi.com/article/10.3390/foods14203488/s1, Figure S1. Ingredients of 8 variants of emulsion; Figure S2. Ingredients of pate; Figure S3. Five variants of pate mass; Figure S4. Heat treatment of canned pâtés.
