# Supplementary material for: Functional and Sensory Properties of Pâtés Formulated with Emulsions from Chicken By-Products
Source: Foods. 2025 Oct 13;14(20):3488. doi: 10.3390/foods14203488 (PMC12564544; doi:10.3390/foods14203488)
Supplement: Supplementary file 1 [file foods-14-03488-s001.zip › foods-3892635-supplementary.pdf]

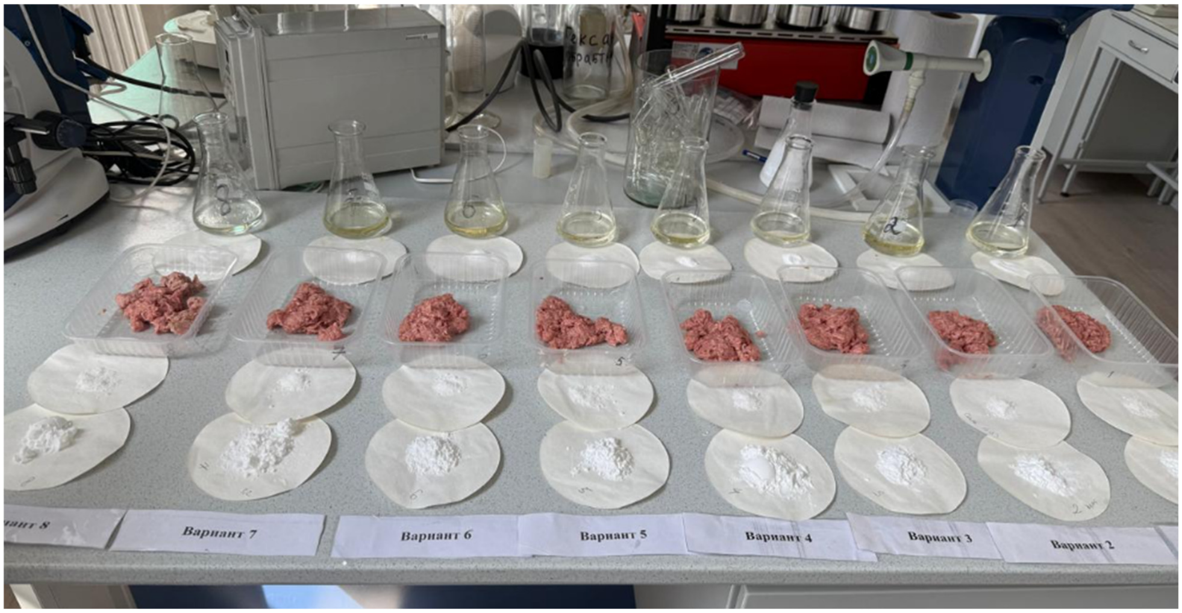

Figure S1. Ingredients of 8 variants of emulsion.

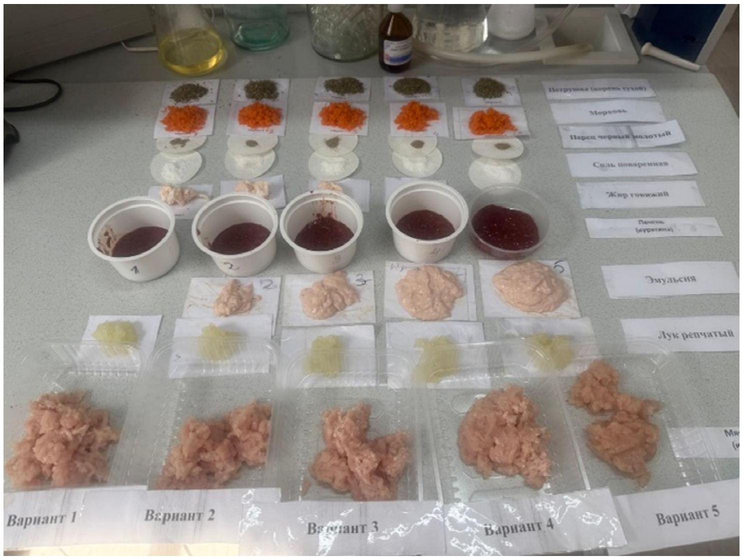

Figure S2. Ingredients of pate.

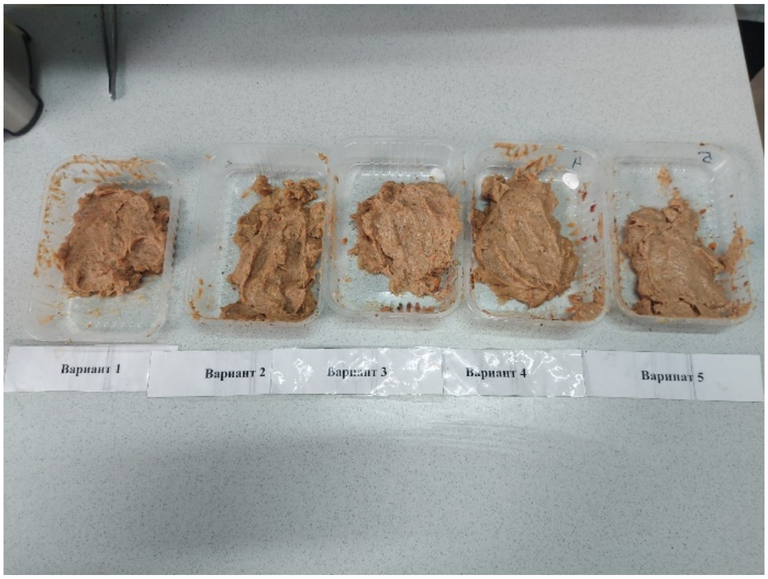

Figure S3. Five variants of pate mass.

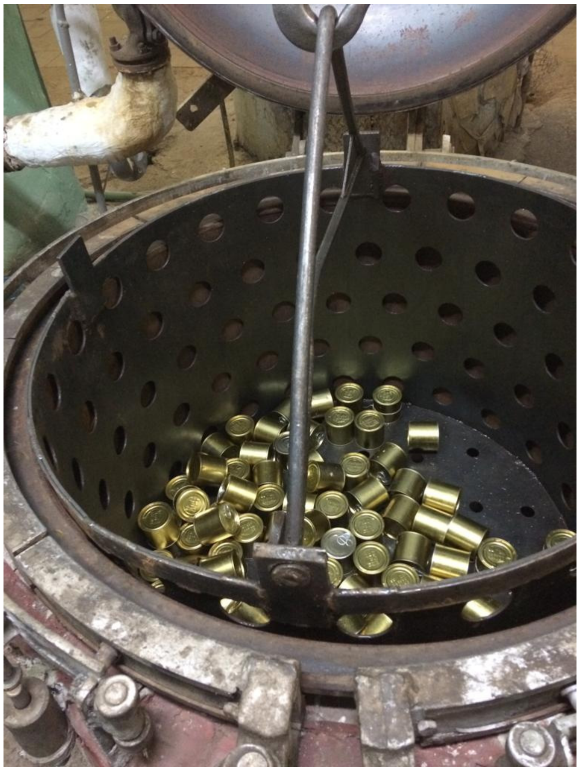

Figure S4. Heat treatment of canned pâtés.
